# Progressive supranuclear palsy’s economical burden: the use and costs of healthcare resources in a large health provider in Israel

**DOI:** 10.1007/s00415-023-11714-1

**Published:** 2023-04-17

**Authors:** Yael Barer, Raanan Cohen, Meital Grabarnik-John, Xiaolan Ye, Jorge Zamudio, Tanya Gurevich, Gabriel Chodick

**Affiliations:** 1 Maccabitech, Maccabi Institute for Research and Innovation, Maccabi Healthcare Services, Ha’Mered 27, 6812509 Tel Aviv, Israel; 2 AbbVie Inc., Hod Hasharon, Israel; 3 AbbVie Inc., North Chicago, IL USA; 4 Tel Aviv Sourasky Medical Center, Tel Aviv, Israel; 5 Sagol School of Neuroscience, Tel Aviv University, Tel Aviv, Israel; 6 Sackler School of Medicine, Tel Aviv University, Tel Aviv, Israel

**Keywords:** Economic burden, Progressive supranuclear palsy, Healthcare services, Medical expenditure, Direct costs

## Abstract

**Background:**

Progressive supranuclear palsy (PSP) is a rare and fatal neurodegenerative movement disorder with no disease modifying therapy currently available. Data on the costs associated with PSP are scarce. This study aims to assess the direct medical expenditure of patients with PSP (PwPSP) throughout disease course.

**Methods:**

This retrospective cohort study is based on the data of a large state-mandated health provider in Israel. We identified PwPSP who were initially diagnosed between 2000 and 2017. Each PwPSP was randomly matched to three health-plan members without PSP by birth-year, sex, and socioeconomic status. Healthcare resources’ utilization and related costs were assessed.

**Results:**

We identified 88 eligible PwPSP and 264 people in the reference group; mean age at diagnosis was 72.6 years (SD = 8.4) and 53.4% were female. The annual direct costs of PwPSP have risen over time, reaching US$ 21,637 in the fifth year and US$ 36,693 in the tenth year of follow-up vs US$ 8910 in the year prior diagnosis. Compared to people without PSP, PwPSP had substantially higher medical expenditure during the years prior- and post-index date.

**Conclusion:**

The present study demonstrates higher economic burden, which increases with time, in PwPSP as compared to those without.

**Supplementary Information:**

The online version contains supplementary material available at 10.1007/s00415-023-11714-1.

## Background

Progressive supranuclear palsy (PSP) is a fatal but rare neurodegenerative disease [1] with an estimated annual prevalence of 0.2–8.3 per 100,000 person [2] and median survival time of 5–8 years [3]. PSP’s prevalence in Maccabi healthcare services (MHS) is 5.3/100,000 [4]. PSP is characterized by various symptoms, such as mobility difficulties (slowness and unsteadiness which lead to frequent falls), cognitive impairment, behavioral changes, dysarthria and dysphagia, and visual manifestations (blurred vision and vertical supranuclear gaze palsy) [5]. There are no disease modifying treatments for PSP, and therefore, with time, those clinical features deteriorate, even under the best symptomatic medical management [6]. Symptomatic therapy includes some pharmacologic therapies: (a) levodopa, dopamine agonists and amantadine for bradykinesia and other motor symptoms; however, effect is usually mild and transient; (b) zolpidem and ophthalmic lubricants for ocular motor deficits and saccadic speed; (c) melatonin, clonazepam, or trazodone for sleep initiation and maintenance; (d) gentle laxatives for constipation; (e) anti-depressants; and (f) botulinum toxin for blepharospasm, eyelid opening apraxia, retrocollis, or sialorrhea. Patients with PSP (PwPSP) also benefit from a multidisciplinary non-pharmacologic therapies including (a) physical therapy to reduce the risk of falls; (b) speech therapy for vocal changes, communication, and safe eating and drinking; (c) occupational therapies for safety at home; and (d) social workers to aid with patients’ and families’ quality of life, especially toward the end of life, and stress [7]. Despite PSP’s rarity, the use of these healthcare services is accompanied by elevated medical expenditure, which might put a significant burden on healthcare systems [8]. To our knowledge, other than one abstract reporting 3 years of follow-up [9], there are no studies longitudinally measure the economic burden in PwPSP throughout disease course, exploring the trajectory of healthcare use and related costs as disease progresses. In addition, we were able to find only one abstract dealing with the comparison of PSP’s economic burden to that of people without PSP [10], in the interest of understanding the excess burden of PSP on the healthcare system. The objective of this study is to assess the direct medical costs of PwPSP compared with matched non-PSP controls from the health provider perspective in Israel.

## Methods

### Study design and patients

This retrospective cohort study was performed using the comprehensive database of the Maccabi Healthcare Services (MHS), a 2.6-million-member state-mandated health plan (payer–provider) in Israel. Membership in MHS is free and open to all Israeli residents. MHS has maintained a computerized database of electronic health records since 1993, containing extensive longitudinal data on a stable population (~ 1% annual turnover).

We identified all MHS members aged 40 or above that had at least 1 documented PSP diagnosis anytime between 2000 and 2017 (inclusive). PSP was documented either using an internal MHS specific code for PSP or an external documented diagnosis using International *Classification of Diseases ninth edition* (ICD-9) code 333.0. Due to ICD-9 = 333.0 unspecific nature, potential cases indicated solely by an external diagnosis were verified by reviewing hospital discharge reports. The index date was defined as the earliest date of PSP diagnosis. Eligible study patients were also required to have at least 1 year of continuous enrollment in MHS prior to the index date (to ensure cases were incident) as well as 1-year post-index date. Each PwPSP was randomly matched by birth-year, sex, and socioeconomic-status (SES) categories (low, medium or high) to MHS members without PSP in a 1:3 ratio. MHS members without PSP were given their respective matched PwPSP’s index date.

All data were anonymous. This study followed the Strengthening the Reporting of Observational Studies in Epidemiology (STROBE) reporting guideline for cohort studies.

### Variables and measurements

#### Demographic variables

MHS's databases were used to collect information on baseline demographics, including age, sex, and SES. SES was based on a score ranked with 1 (lowest) to 10 derived for commercial purposes by Points Location Intelligence^®^ using data on patients’ residential area. This score is highly correlated with SES measured by the Central Bureau of Statistics [11]. SES was categorized into low (1–4), medium (5–6), and high (7–10). Populations’ comorbidities were assessed using MHS chronic disease registries (i.e., cardiovascular disease [12], diabetes [13], hypertension [14], chronic kidney disease [15], mild cognitive decline [16], dementia [16], and cancer [17]).

#### PSP’s economic burden

Two analyses were performed to assess the healthcare resource utilization (HCRU) and the economic burden of PSP. First, we analyzed the long-term burden manifested by the annual HCRU and its related costs from 1 year prior to the index date until end of follow-up. HCRU was defined as all hospitalizations days, emergency department (ED) visits, outpatient visits, treatments (procedures and tests), and medications dispensations. End of follow-up was defined as the earlier of (1) death date, (2) MHS leave date, (3) 10 years post-diagnosis, or (4) end of study—December 31, 2018. Second, we compared the HCRU and its related costs between PwPSP and randomly matched MHS members without PSP during 1 year prior- and 1 year post-index.

Direct costs were extracted from the ministry of health’s January 2019 price list [18] and presented in US Dollars (USD) using the Purchasing Power Parities (PPP) of 2018 as a conversion factor [1 USD = 3.663 Israeli shekels]. Discount for cost of different years was not taken into account and a fixed price was considered.

### Statistical methods

Descriptive statistics are presented using frequencies and proportions for categorical variables and mean values with standard deviations (SD) or medians with interquartile range (IQR) as appropriate. Baseline differences between groups were assessed using Chi-square test and two independent samples *T* test or non-parametric test, as appropriate.

PSP's economic burden manifested as annual, per patient HCRU and direct costs presented as estimated means (EM, and 95% confidence interval [CI]). In cases where the last year of follow-up was less than 12 months, data were annualized. The SD of annualized cost in the last year was plotted versus the number of months of the same, incomplete, year [19]. Pearson correlation was assessed and the results suggest that the SD for the annualized cost is fairly independent of the number of months of follow-up (*R* = 0.29, *p* = 0.379, data not shown). For the long-term burden analysis, generalized linear mixed models (GLMMs) were used. For the comparison between PwPSP and MHS members without PSP using generalized Estimating Equations (GEE) were used, with matched subjects defined as repeated measurements and correlation matrix defined as exchangeable. HCRU were considered to have a negative binomial distribution with log link and cost data were considered to have a gamma distribution with log link and top 1% of data were excluded from the analysis to exclude sever oncologic patients. Patients without any use of a specific healthcare resource were excluded from each specific analysis. Adjustments for baseline characteristics and comorbidities were made when needed. In addition, each healthcare resource costs for the both populations were summed and plotted to compare the overall costs per year. Since we matched in a 1:3 ratio, the costs of the non-PSP cohort were divided by 3. All analyses were performed in SPSS 27 (IBM-SPSS Inc., Armonk, NY). Figures were created using ‘ggplot2’ package in R software version 4.02.

## Results

### Patients’ characteristics

We identified and matched 88 eligible PwPSP to 264 MHS members without PSP; mean age at index date was 72.6 years (SD = 8.3) and 53.4% were female. Baseline characteristic were similar between cohorts, apart from prevalence of dementia (PwPSP: 29.5% vs. patients without PSP: 2.5%) and mild cognitive impairment (PwPSP: 14.8% vs. patients without PSP: 0.4%), which were significantly higher among PwPSP (*p* < 0.001) (Table 1).

**Table 1:** Baseline characteristics PwPSP and MHS members without PSP

	PSP (*n* = 88)	No PSP (*n* = 264)	*p* value^a^
Age at index, years			
Mean (SD)	72.6 (8.4)	72.6 (8.4)	0.991
Sex, female			
Yes (%)	47 (53.4)	141 (53.4)	1
Socioeconomic status			
Low (%)	7 (8.0)	21 (8.0)	1
Medium (%)	30 (34.1)	90 (34.1)	
High (%)	46 (52.3)	138 (52.3)	
Missing (%)	5 (5.7)	15 (5.7)	
Smoking status			
Current (%)	4 (4.5)	25 (9.5)	0.460
Past (%)	3 (3.4)	9 (3.4)	
Never (%)	74 (84.1)	204 (77.3)	
Missing (%)	7 (8.0)	26 (9.8)	
Cardiovascular disease			
Yes (%)	30 (34.1)	102 (38.6)	0.446
Diabetes mellitus			
Yes (%)	18 (20.5)	59 (22.3)	0.710
Hypertension			
Yes (%)	59 (67.0)	172 (65.2)	0.746
Chronic kidney disease			
Yes (%)	54 (61.4)	130 (49.2)	0.049
Cancer			
Yes (%)	21 (23.9)	44 (16.7)	0.132
Dementia			
Yes (%)	26 (29.5)	6 (2.3)	< 0.001
Mild cognitive impairment			
Yes (%)	13 (14.8)	1 (0.4)	< 0.001

*PwPSP* patients with progressive supranuclear palsy, *SD* standard deviation

^a^P values were calculated using Chi-square test for categorical variables and independent t test for continuous variables

Overall, one PwPSP (1.1%) and 14 (5.3%) of the reference group left MHS during the study period.

### Long-term economic burden of PwPSP

**Fig. 1:**
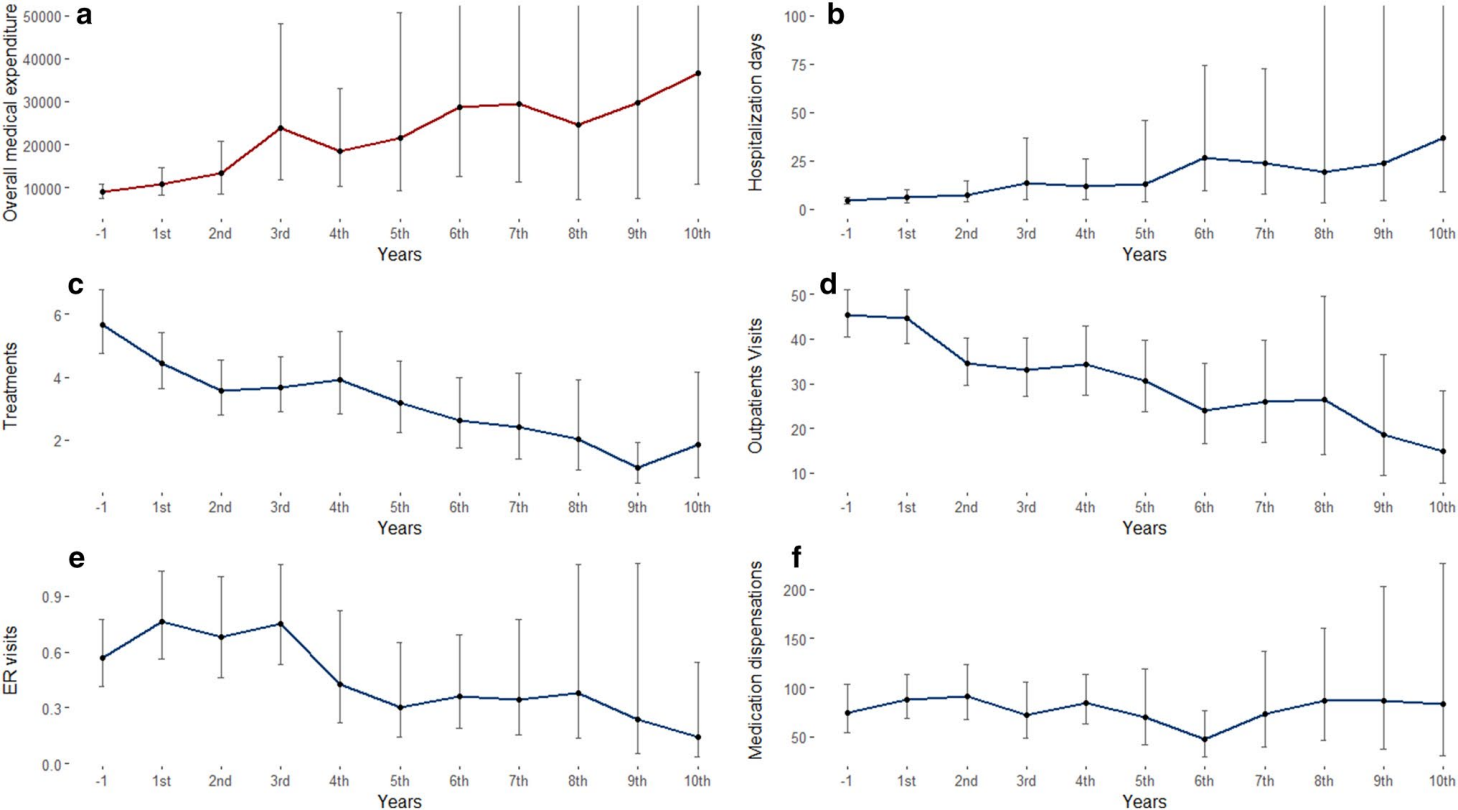
Estimated mean and 95%CI of HCRU and costs throughout disease course. **a** Overall medical expenditure, **b** number of hospitalization days, **c** number of treatments, **d** number of outpatients’ visits, **e** number of emergency department visits, and **f** number of medication packs

Throughout the 10-years study follow-up period, an increase of overall direct costs was observed (Fig. 1a). Overall costs during the tenth year after diagnosis was more than four times the costs in the year preceding first diagnosis (US$ 36,693 [95% CI 10,729–125,490] *n* = 16, US$ 8910 [95% CI 7379–10,759] *n* = 88, respectively). Even when comparing the cost increase in the fifth year (*n* = 42), there is a 2.5-fold increase of costs (US$ 21,637 [95% CI 9218–50,788]). While, an increase of number of hospitalizations days was observed (Fig. 1b, from 4.1 days [95% CI 2.7–6.6] in the year prior index date to 36.8 days [95% CI 8.9–151.6] in the tenth year post-index date), a decrease in use of treatments, outpatients’ clinics, and ED was observed (Fig. 1panels c–e). There was no change in the number of medication dispensations per patient (Fig. 1f). The numbers of patients with at least one use in each healthcare resource are shown in Online Resource 2.

Estimated means (95% CI) were calculated using GLMM for each year of follow-up from the last year prior to PSP first diagnosis (“− 1”) to the 10th year post-diagnosis. For each year, the model included only patients with at least one event. Note, each panel has a different Y axis.

### Comparison of economic burden, PwPSP vs. reference group

During 1-year prior index date (Table 2), compared with MHS members without PSP, higher proportion of PwPSP were hospitalized (PwPSP: 40% vs. patients without PSP: 15.9%); however, among those with at least one hospitalizations, the length of stay was similar between cohorts. A higher proportion of PwPSP had at least one ED visit (38.6%) compared to those without PSP (13.6%). PwPSP had more visits to outpatient clinics (adjusted EM [95% CI], PwPSP: 48.8 [41.2–57.8] vs. patients without PSP: 26.1 [21–32.4]) and purchased more medications (PwPSP: 104.8 [85.4–128.7] vs. patients without PSP: 70.8 [55.1–91]) compared to MHS members without PSP. In line with these results, the corresponding costs of outpatient visits and medication dispensations as well as outpatient treatment and procedures were higher in PwPSP compared to those without. Finally, the overall direct costs during the year preceding PSP diagnosis were approximately twofold higher among PwPSP compared to those without PSP (adjusted EM [95% CI], PwPSP: US$ 9128 [6994–11,914] vs. patients without PSP: US$ 4316 [3214–5796]).

**Table 2:** HCRU^a^ and costs^b^ in PwPSP and matched MHS members without PSP during the year prior index date

	N with event					Crude estimated mean			Adjusted estimated mean^c^		
	PSP *n* = 88	No PSP *n* = 264	*p* value crude	*p* value adjusted		PSP	No PSP	*p* value	PSP	No PSP	*p* value
Hospitalization days	36 (40.9%)	42 (15.9%)	< 0.001	< 0.001	HCRU	10.1 (7.3–13.9)	10.9 (7.4–16.1)	0.746	10 (5.7–17.5)	11.9 (5.5–25.6)	0.529
					Costs	8312 (6073–11,377)	6576 (4710–9180)	< 0.001	7371 (4492–12,096)	6438 (3149–13,163)	0.592
ED visits	34 (38.6%)	36 (13.6%)	< 0.001	< 0.001	HCRU	1.5 (1.3–1.7)	1.4 (1.2–1.6)	0.629	1.2 (1–1.6)	1.1 (0.8–1.3)	0.192
					Costs	314 (277–356)	301 (268–337)	0.612	332 (283–389)	287 (232–356)	0.097
Outpatients visits	88 (100.0%)	250 (94.7%)	0.997	0.996	HCRU	45.5 (40.5–51.1)	24.3 (21.9–27.1)	< 0.001	48.8 (41.2–57.8)	26.1 (21–32.4)	< 0.001
					Costs	3205 (2866–3584)	1746 (1585–1922)	< 0.001	3267 (2791–3825)	1737 (1426–2116)	< 0.001
Treatment and procedures	83 (94.3%)	221 (83.7%)	0.017	0.051	HCRU	6 (5.1–7.1)	7.4 (5.4–10.1)	0.259	6.5 (4.7–8.9)	7.1 (4.7–10.7)	0.568
					Costs	1561 (1089–2238)	962 (796–1163)	0.020	1730 (965–3099)	957 (495–1848)	0.006
Medications	86 (97.7%)	243 (92.0%)	0.080	0.274	HCRU	112.6 (98.4–128.8)	79.6 (71.3–88.8)	< 0.001	104.8 (85.4–128.7)	70.8 (55.1–91)	< 0.001
					Costs	1262 (1063–1498)	848 (736–977)	< 0.001	1105 (858–1422)	722 (537–972)	< 0.001
Overall direct costs					Costs	9449 (7926–11,263)	4881 (4163–5722)	< 0.001	9128 (6994–11,914)	4316 (3214–5796)	< 0.001

Estimated means, 95% CI, and p value were extracted from GEE with negative binomial distribution with log link for HCRU analyses and gamma distribution with log-link function for costs analyses

*PwPSP* patients with progressive supranuclear palsy, *HCRU* healthcare resource utilization, *ED* emergency department, *CI* confidence interval, *GEE* generalized estimating equations

^a^Presented data were calculated for patients with ≥ 1 HCRU

^b^Top 1% was excluded

^c^Analyses were adjusted for age, sex, socioeconomic status, chronic kidney disease, dementia, and mild cognitive impairment

In the year post-index date (Table 3), PwPSP were significantly more likely to be hospitalized and visit the ED, and have a lower use of outpatient treatment and procedures. Finally, the overall direct costs during 1 year after PSP diagnosis were approximately 1.5-fold higher among PwPSP compared to those without PSP (adjusted EM [95% CI], PwPSP: US$ 12,020 [8643–16,716] vs. patients without PSP: US$ 7258 [5041–10,449]).

**Table 3:** HCRU^a^ and costs^b^ in PwPSP and matched MHS members without PSP during the year post-index date

	N with event					Crude estimated mean			Adjusted estimated mean^c^		
	PSP *n* = 88	No PSP *n* = 264	*p* value crude	*p* value adjusted		PSP	No PSP	*p* value	PSP	No PSP	*p* value
Hospitalization days	32 (36.4%)	45 (17.0%)	< 0.001	0.028	HCRU	16.3 (10.4–25.5)	14.9 (7.5–29.7)	0.840	11.6 (6.3–21.3)	8.7 (4.3–17.7)	0.402
					Costs	10,085 (6621–15,361)	8292 (6056–11,353)	0.465	10,275 (5752–18,353)	6787 (3404–13,534)	0.239
ED visits	37 (42.0%)	50 (18.9%)	0.385	0.006	HCRU	1.1 (0.7–1.7)	0.6 (0.4–1)	0.084	1.9 (1.4–2.4)	1.3 (0.9–1.8)	0.012
					Costs	383 (328–447)	304 (271–341)	0.018	379 (314–457)	308 (233–406)	0.096
Outpatients visits	87 (98.9%)	245 (92.8%)	0.065	0.163	HCRU	45.2 (39.6–51.5)	27.7 (24.7–31.1)	< 0.001	44.6 (36.3–54.8)	26.8 (21.3–33.7)	< 0.001
					Costs	2982 (2658–3346)	1968 (1800–2152)	< 0.001	3007 (2517–3591)	1917 (1562–2353)	< 0.001
Treatment and procedures	82 (93.2%)	226 (85.6%)	0.069	0.104	HCRU	4.8 (3.9–5.8)	7.3 (5.9–8.9)	0.003	5.6 (4.1–7.7)	8 (5.3–12)	0.017
					Costs	758 (520–1105)	1293 (1034–1615)	0.017	750 (434–1298)	1210 (756–1937)	0.027
Medications	85 (96.6%)	244 (92.4%)	0.182	0.602	HCRU	126.6 (111–144.3)	83.5 (74.6–93.4)	< 0.001	108.4 (87.8–133.8)	67.4 (52.4–86.6)	< 0.001
					Costs	1451 (1242–1695)	963 (832–1115)	< 0.001	1215 (953–1548)	794 (602–1049)	< 0.001
Overall direct costs					Costs	11,198 (9245–13,563)	6801 (5825–7941)	< 0.001	12,020 (8643–16,716)	7258 (5041–10,449)	< 0.001

Estimated means, 95% CI, and p value were extracted from GEE with negative binomial distribution with log link for HCRU analyses and gamma distribution with log-link function for costs’ analyses

*PwPSP* patients with progressive supranuclear palsy, *HCRU* healthcare resource utilization, *ED* emergency department, *CI* confidence interval, *GEE* generalized estimating equations

^a^Presented data were calculated for patients with ≥ 1 HCRU

^b^Top 1% was excluded

^c^Analyses were adjusted for age, sex, socioeconomic status, chronic kidney disease, dementia, and mild cognitive impairment

**Fig.2:**
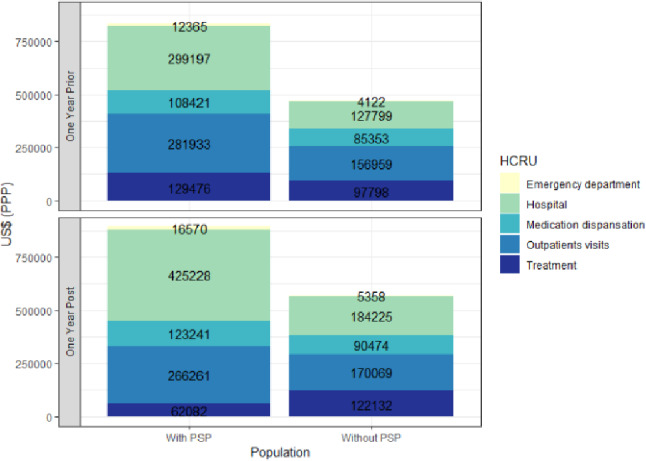
Overall direct costs in the PSP cohort compared with the non-PSP cohort^a^ 1 year prior and 1-year post-index date. ^a^Costs in the non-PSP cohort were divided by 3 to match the number of patients in the PSP cohort

Figure 2 demonstrates the overall direct costs by type of HCRU for the whole PSP cohort compared with the non-PSP cohort. During both years, the difference between population was mainly derived by higher hospitalization and outpatient clinic costs.

## Discussion

This retrospective cohort study utilized a clinical database spanning over two decades to quantify the HCRU and direct costs associated with PSP. It is well established that PSP is a progressive disease, where gait, swallowing, and speech functions deteriorate with time [6]. To improve the understanding of longitudinal utilization patterns, we examine the annual HCRU and related costs among PwPSP, up to 10 years after diagnosis.

Our results show that PSP is associated with a substantial direct medical costs that quadrupled between the year prior diagnosis and the tenth year after diagnosis, indicating that a greater use of services that PwPSP require over time. Throughout a period of 10 years after diagnosis, there is a cumulative added cost of US$ 148,000 per patient.

Data on the direct medical costs of PSP are scarce. Our findings are in line with two previous publications assessing the economic burden of PSP, demonstrating that disease severity is a significant contributor to the increase of direct costs [8, 20]; the semiannual costs increased from €9290 in patients in early stages (1–2, measured by the Unified MSA Rating Scale, Part IV) to €20,630 in patients in advanced stages (4–5) [20]. Similar trends are shown in France, Germany and the UK where disease severity was measured using Parkinson Plus Symptoms scale [8]. A recently published abstract reported a 10% increase of number of hospitalizations per year, for 3 year follow-up [9]. We observed a 30% increase in length of hospital stay per year. One publication addressed the economic burden associated with frontotemporal degeneration, while 5.4% of patients were defined as either PSP or corticobasal syndrome, the economic burden was not reported for that group [21]. Due to the scarcity of studies in the field, we compared this study’s findings to those of other neurodegenerative diseases: Alzheimer’s disease (AD)—another tau based neurodegenerative disease, and PD, the most common misdiagnosis in PwPSP [4, 22]. Both conditions demonstrated an increase of medical expenditure with disease progression [23–30]. Whereas PD's direct costs were approximately US$ 6000 for patients with mild–moderate PD and US$ 7400 [29] for patients with advanced disease (median disease duration: 4 years), and PSP’s direct costs were approximately US$ 10,000 and US$ 18,000 at the first diagnosis and fourth year after diagnosis, respectively. While PSP’s medical expenditure continued to increase throughout disease course, the current study observed a decrease in community-based HCRU and an increase in length of hospital stay. This discrepancy might be explained by the understanding of the Israeli health system; In Israel, when the more severe patients are relocating to nursing homes or assisted living facilities are not reimbursed by MHS and therefore were not captured. Similar observation was reported in a study in Germany [26] where HCRU of patients with AD living at home were compared with those of institutionalized patients. The number of outpatient and treatments as well as related costs were lower in institutionalized patients, while the number of hospitalization and related costs were higher in these patients [26].

Overall, during 1 year prior- and post-index date, when compared with people without PSP, PwPSP had higher HCRU. Direct medical expenditure per patient and that for the whole cohort were higher in PwPSP during both years. These findings resemble those of a recent published abstract [10]. In addition, these findings are in line with those of resembling conditions; AD [31] and PD [27, 32, 33]. The higher costs during the year prior index can be explained by the known delayed in PSP’s diagnosis [4], and by higher HCRU leads to the diagnosis.

The present study has several limitations. First, the analysis was conducted from the healthcare system perspective, and therefore, we focused on direct medical expenditure. Direct costs of services provided beyond the framework of the MHS or indirect costs, such as home renovation, nursing home, loss of caregiver income, and unpaid care, were not included. However, when excluding the unpaid costs in a study reporting cross-sectional costs analysis (mean PSP duration ranges 3.9–4.4), the mean direct annual costs (calculated by [total semiannual costs – the unpaid care]*2) is approximately €16,000, similar to our corresponding fourth year costs; US$ 18,358 [8]. In addition, it has been shown that unpaid family care accounts for most of the cost of PSP [8]; thus, we may have underestimated the overall economic burden of disease. However, given the low copayment rates in Israel, it can be assumed that the majority of patients received mostly MHS-subsidized treatments. Second, discount for cost of different years was not taken into account and a fixed price was considered. However, we believe that this method serves the study objective better. In the long term, we wanted to explore the change in medical costs through disease course, and thus, changes caused by currency or price changes would have register as changes due to the disease and cause a misleading conclusion. For the comparison between PSP and non-PSP cohorts, since the matched MHS members were given their respective PwPSP index date, prices were identical per event. Third, follow-up time was lower in PwPSP compared with MHS members without PSP. However, the comparison of economic burden between study populations was assessed for 1 year—before and after—index date, thus ensuring 100% of patients in the analysis. Notwithstanding, this requirement might cause an exclusion of the most severe patients who might have died during that year. Despite this possible selection bias toward the less severe cases (which might have caused an underestimate of economic burden), we were able to show a significant difference in the economic burden compared to people without PSP. Forth, it is known that some PSP phenotypes may be more severe; however, we were not able to differentiate between PSP’s different phenotypes. In addition, it is known that PwPSP surviving 10 years post-diagnosis are most likely the less severe cases [34]; still, we were able to observe an increased direct medical expenditure. Fifth, we were unable to extract the cause of admission to the hospital to understand and help a more targeted supportive care. Additional limitations are those inherit to database research. First, we identified PwPSP only by one diagnosis, which may cause misclassification of the disease; however, in a previous analysis of PSP’s epidemiology, using the same criteria, we showed that our results are in line with the other publications [4]. The consequence of such non-differential misclassification of PSP is dilution of the measure of effect size and underestimation of the true economic burden of the disease due to “bias towards the null”. Second, despite adjustment of the models, residual confounding by unmeasured variables cannot be excluded. One such important unmeasured confounders is low educational attainment that was found as a major risk factor for PSP [35]. Lower educational level and poorer health literacy have been association with lower use of healthcare services, primarily of digital services in Israel [36]. Therefore, the true economic burden of PSP might have been greater than calculated in our analysis. This study has several strengths. First, to our knowledge, this is the first study assessing the economic burden associated with PSP in such comprehensive manner. Second, our use of routinely captured clinical database allowed for long study period, with some patients followed for more than 10 years.

## Conclusions

Compared with individuals without PSP, PwPSP have considerably higher use of healthcare services and greater associated direct medical costs accruing from around the time of diagnosis throughout disease progression. PSP’s direct costs also increase with time. Investing in disease modifying therapies’ clinical trials nowadays might reduce the economic burden for this population and the healthcare system in the long term.

## Supplementary Information

Below is the link to the electronic supplementary material.

Supplementary file1 (PDF 405 KB)

## Data Availability

Data sharing is not applicable in this research article due to privacy matters, and therefore, it was not approved by the MHS internal review board.
