# Supplementary material for: Progressive supranuclear palsy’s economical burden: the use and costs of healthcare resources in a large health provider in Israel
Source: J Neurol. 2023 Apr 17;270(8):3770–8. doi: 10.1007/s00415-023-11714-1 (PMC10345081; doi:10.1007/s00415-023-11714-1)
Supplement: Supplementary file 1 — Supplementary file1 (PDF 405 KB) [file 415_2023_11714_MOESM1_ESM.pdf]

Supplementary Information (SI)

Progressive Supranuclear Palsy's Economical Burden; The use and costs of Healthcare Resources in a large health provider in Israel

Journal of Neurology

Yael Barer MSc[1], Raanan Cohen MD, MBA[2], Meital Grabarnik-John DVM[2], Xiaolan Ye PhD[3], Jorge Zamudio MD[3], Tanya Gurevich MD[4-6], Gabriel Chodick PhD[1,6]

Affiliations:

[1]Maccabitech, Maccabi Institute for Research and Innovation, Israel

[2]AbbVie Inc., Hod Hasharon, Israel

[3]AbbVie Inc., North Chicago, IL, USA

[4]Tel Aviv Sourasky Medical Center, Israel

[5]Sagol School of Neuroscience, Tel Aviv University

[6]Sackler School of Medicine, Tel Aviv University

Corresponding Author:

Yael Barer

Ha'Mered 27, Tel Aviv, Israel 6812509

E-mail: Barer_y@mac.org.il

Tel: +975-3-7462762

Online Resource 1: Definitions of Dementia and Mild cognitive impairment

- **Dementia**: using the cognitive disorders registry:
    - High certainty: at least 2 visit diagnoses or 1 active diagnosis by geriatrician/psychiatrist/neurologist or at least 2 visit diagnoses by general physician & sweet 16≤13, or at least 1 active diagnosis by general physician & sweet 16≤13, or at least 1 diagnosis from special medication approvals.
    - Low certainty: at least 2 visit diagnoses by general physician without sweet 16≤13 or 1 active diagnosis by general physician without sweet 16≤13, or at least 2 purchases of medications within 90 days.
- **Mild cognitive impairment**: using the cognitive disorders registry:

At least 2 visit diagnoses by general physician & sweet 16 (Mini Mental state examination =14-15 or 1 active diagnosis by general physician & sweet 16=14-15.

Online Resource 2: Number and percentage of PwPSP in the analysis, by year prior/post first diagnosis date and type of healthcare resource

	PwPSP in follow-up	PwPSP with hospitalization	PwPSP with ED visit	PwPSP with treatment	PwPSP with outpatient visit	PwPSP with medication purchase
**1 year prior**	88 (100.0%)	36 (40.9%)	34 (38.6%)	83 (94.3%)	88 (100.0%)	86 (97.7%)
**1 year post**	88 (100.0%)	32 (36.4%)	37 (42.0%)	82 (93.2%)	87 (98.9%)	85 (96.6%)
**2 years post**	86 (97.7%)	36 (41.9%)	31 (36.0%)	72 (83.7%)	86 (100%)	86 (100.0%)
**3 years post**	71 (80.7%)	37 (52.1%)	29 (40.8%)	61 (85.9%)	70 (98.6%)	71 (100.0%)
**4 years post**	58 (65.9%)	28 (48.3%)	13 (22.4%)	51 (87.9%)	58 (100.0%)	58 (100%)
**5 years post**	42 (47.7%)	17 (40.5%)	7 (16.7%)	32 (76.2%)	38 (90.5%)	42 (100.0%)
**6 years post**	36 (40.9%)	13 (36.1%)	9 (25.0%)	27 (75.0%)	31 (86.1%)	35 (97.2%)
**7 years post**	28 (31.8%)	13 (46.4%)	6 (21.4%)	21 (75.0%)	25 (89.3%)	27 (96.4%)
**8 years post**	21 (23.9%)	7 (33.3%)	5 (23.8%)	13 (61.9%)	18 (85.7%)	19 (90.5%)
**9 years post**	15 (17.0%)	5 (33.3%)	2 (13.3%)	10 (66.7%)	13 (86.7%)	15 (100%)
**10 years post**	14 (15.9%)	3 (21.4%)	2 (14.3%)	8 (57.1%)	11 (78.6%)	13 (92.9%)

PwPSP= patients with Progressive supranuclear palsy, ED= emergency department
